# Supplementary material for: Lamin B1 mapping reveals the existence of dynamic and functional euchromatin lamin B1 domains
Source: Nat Commun. 2018 Aug 24;9:3420. doi: 10.1038/s41467-018-05912-z (PMC6109041; doi:10.1038/s41467-018-05912-z)
Supplement: Supplementary file 1 — Supplementary Information [file 41467_2018_5912_MOESM1_ESM.pdf]

Lamin B1 mapping reveals the existence of dynamic and functional euchromatin lamin B1 domains

Supplementary Information

Laura Pascual-Reguant[1], Enrique Blanco[2], Silvia Galan[2,4], François Le Dily[2,3], Yasmina Cuartero[2,4], Gemma Serra-Bardenys[1,3], Valerio Di Carlo[2], Ane Iturbide[5], Joan Pau Cebrià-Costa[1], Lara Nonell[6], Antonio García de Herreros[3,7], Luciano Di Croce[2,8], Marc A. Marti-Renom[2,3,4,8] & Sandra Peiró[1]

[1]Vall d'Hebron Institute of Oncology, 08035 Barcelona, Spain; [2]Centre for Genomic Regulation (CRG), The Barcelona Institute of Science and Technology, Dr. Aiguader 88, Barcelona, Spain; [3]Departament de Ciències Experimentals i de la Salut, Universitat Pompeu Fabra (UPF), 08003 Barcelona, Spain; [4]Structural Genomic Group, CNAG-CRG, Centre for Genomic Regulation (CRG), The Barcelona Institute of Science and Technology, Baldiri Reixac 4, Barcelona, Spain; [5]Institute of Epigenetics and Stem Cells, D-81377 München, Germany; [6]Servei d'Anàlisi de Microarrays Institut Hospital del Mar d'Investigacions Mèdiques, Barcelona, Spain; [7]Programa de Recerca en Càncer, Institut Hospital del Mar d'Investigacions Mèdiques, Barcelona, Spain; [8]ICREA, Pg. Lluis Companys 23, Barcelona, Spain

Corresponding author: Sandra Peiró. VHIO, C/ Natzaret 115-117, 08035, Barcelona, Spain.
Email: speiro@vhio.net

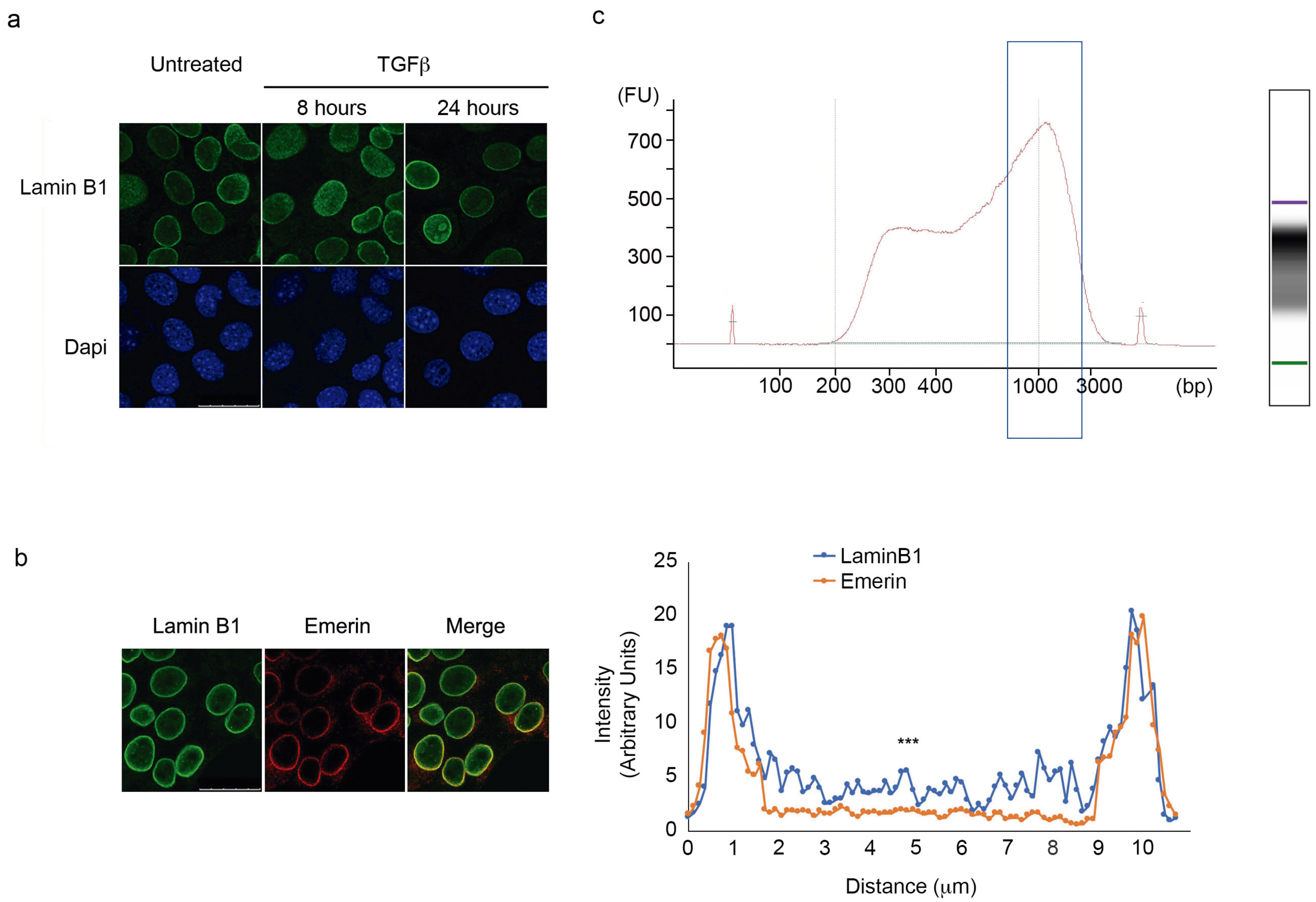

Supplementary Figure 1. Lamin B1 localization at the nuclear interior and bioanalyzer intensity profile. **a** NMuMG cells treated with TGF-β were immunolabelled with laminB1 antibody or stained with DAPI. Scale bar, 25 m. **b** NMuMG cells in untreated conditions were immunolabelled with lamin B1 and emerin antibodies. The colocalization of both signals is shown in the third column as a merge. Scale bar, 25 m (left). All images were obtained by confocal microscopy and are representative of at least three independent replicates. A graphic representation of the antibody signal intensities among the nuclei is shown. Regions of higher intensity belong to the nuclear envelope, and regions of lesser intensity, to the nucleoplasm (right). Data are shown as means of nine cells and were obtained from two technical replicates. ***, significant differences between both signals ($p < 0.001$) was assessed by the two-tailed unpaired Student's t-test. **c** Representative bioanalyzer intensity profile of a lamin B1 ChIP sample. Fragments excluded from the sequencing are within the blue rectangle

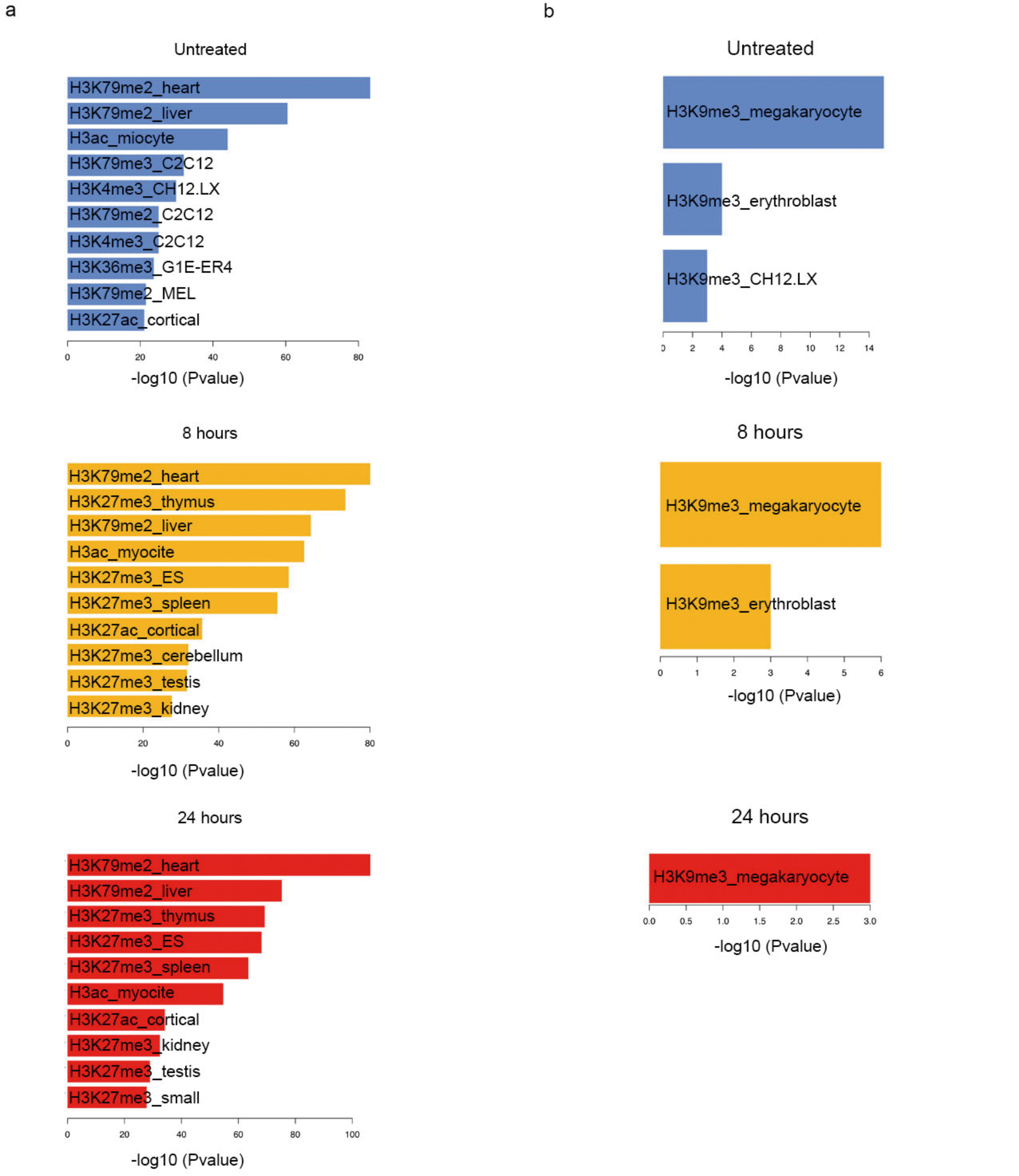

Supplementary Figure 2. Term enrichment analysis of histone marks associated with lamin B1 + sites. a Functional analysis of lamin B1+ genes showing the statistically significant association with histone marks in NMuMG cells that were untreated (blue) or treated with TGF-β for 8 h (orange) or 24 h (red). **b** The same analysis as in (a) was applied to genes not associated with lamin B1 sites under the same conditions: untreated (blue) or treated 8 h (orange) or 24 h (red) with TGF-β.

a

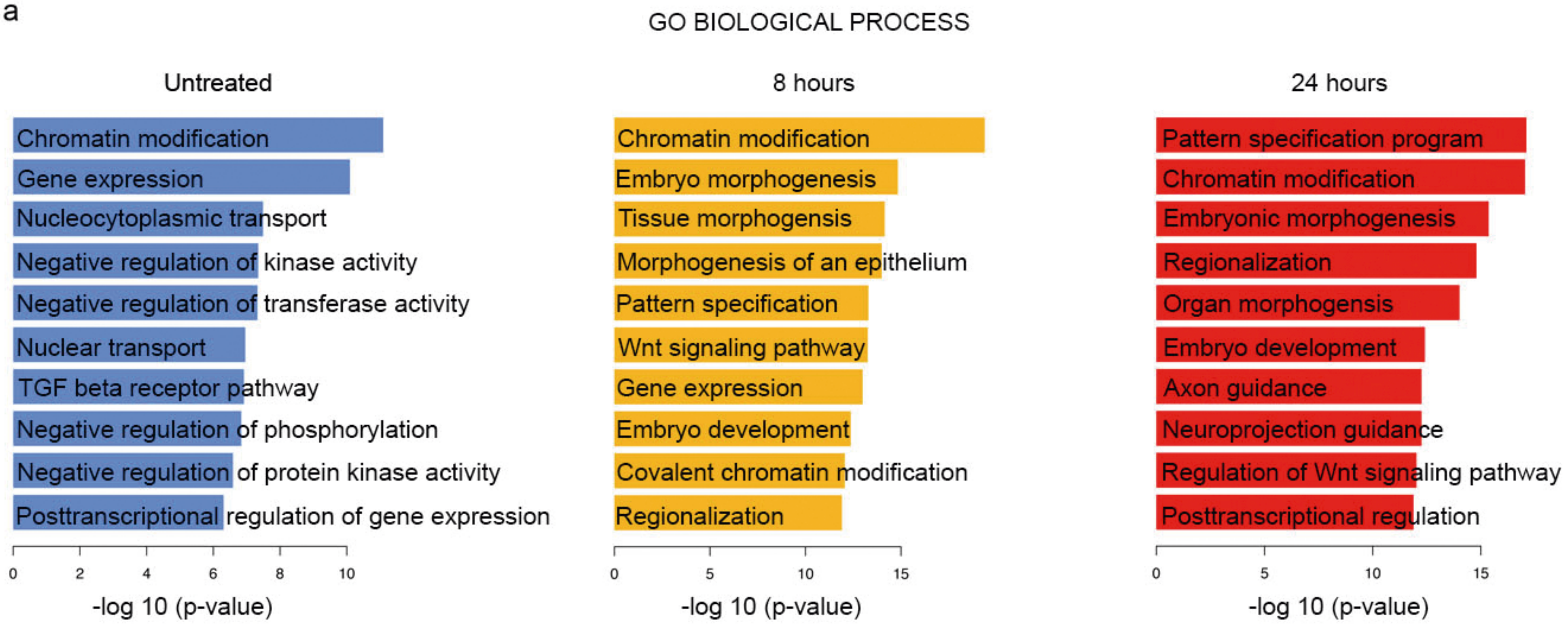

b

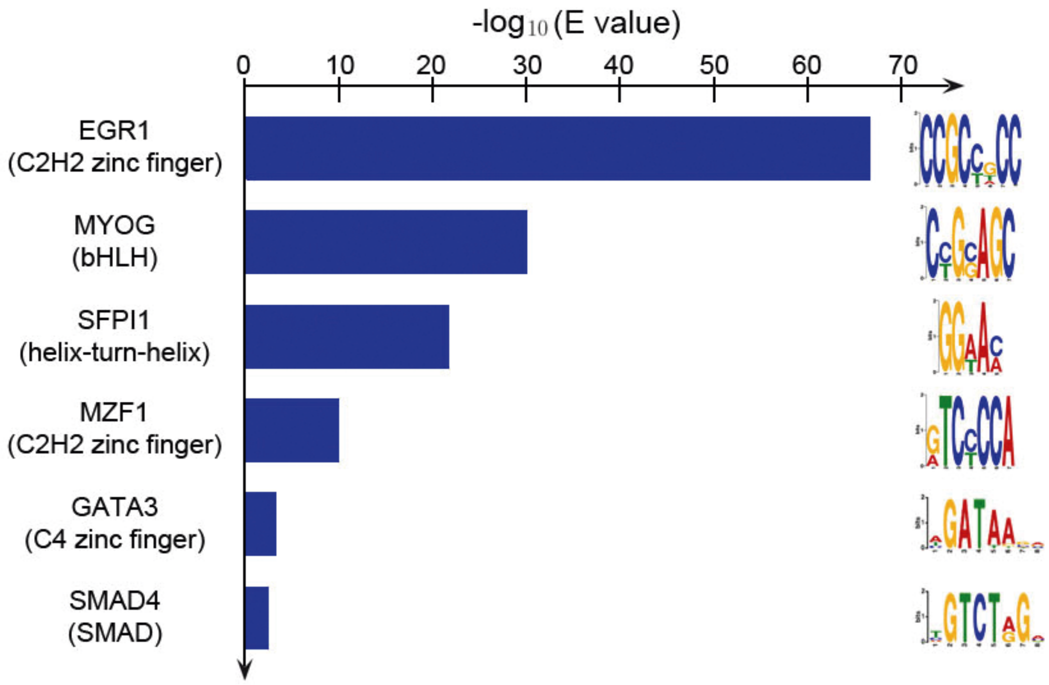

c

Gene Set Name [# Genes (K)]	Description	p-value	FDR q-value
REACTOME_DEVELOPMENTAL_BIOLOGY [396]	Genes involved in Developmental Biology	2.38 e-5	2.12 e-2
BIOCARTA_GATA3_PATHWAY [16]	GATA3 participate in activating the Th2 cytokine genes expression	8.8 e-5	3.92 e-2
BIOCARTA_CALCINEURIN_PATHWAY [21]	Effects of calcineurin in Keratinocyte Differentiation	1.54 e-4	4.56 e-2
REACTOME_SMAD2_SMAD3_SMAD4_HETEROTRIMER_REGULATES_TRANSCRIPTION [27]	Genes involved in SMAD2/SMAD3:SMAD4 heterotrimer regulates transcription	2.56 e-4	4.9 e-2
REACTOME_MYOGENESIS [28]	Genes involved in Myogenesis	2.75 e-4	4.9 e-2

Supplementary Figure 3. Term enrichment analysis of lamin B1 + sites. **a** Functional analysis of statistically significant biological processes of lamin B1+ genes in NMuMG cells untreated (blue) or treated with TGF-β for 8 h (orange) or 24 h (red). **b** Statistically significant transcription factor motif analysis of 5 896 de novo lamin B1+ genes. **c** Signature of pathways associated to the list of previously identified transcription factors. Only statistically significant signature pathways are shown.

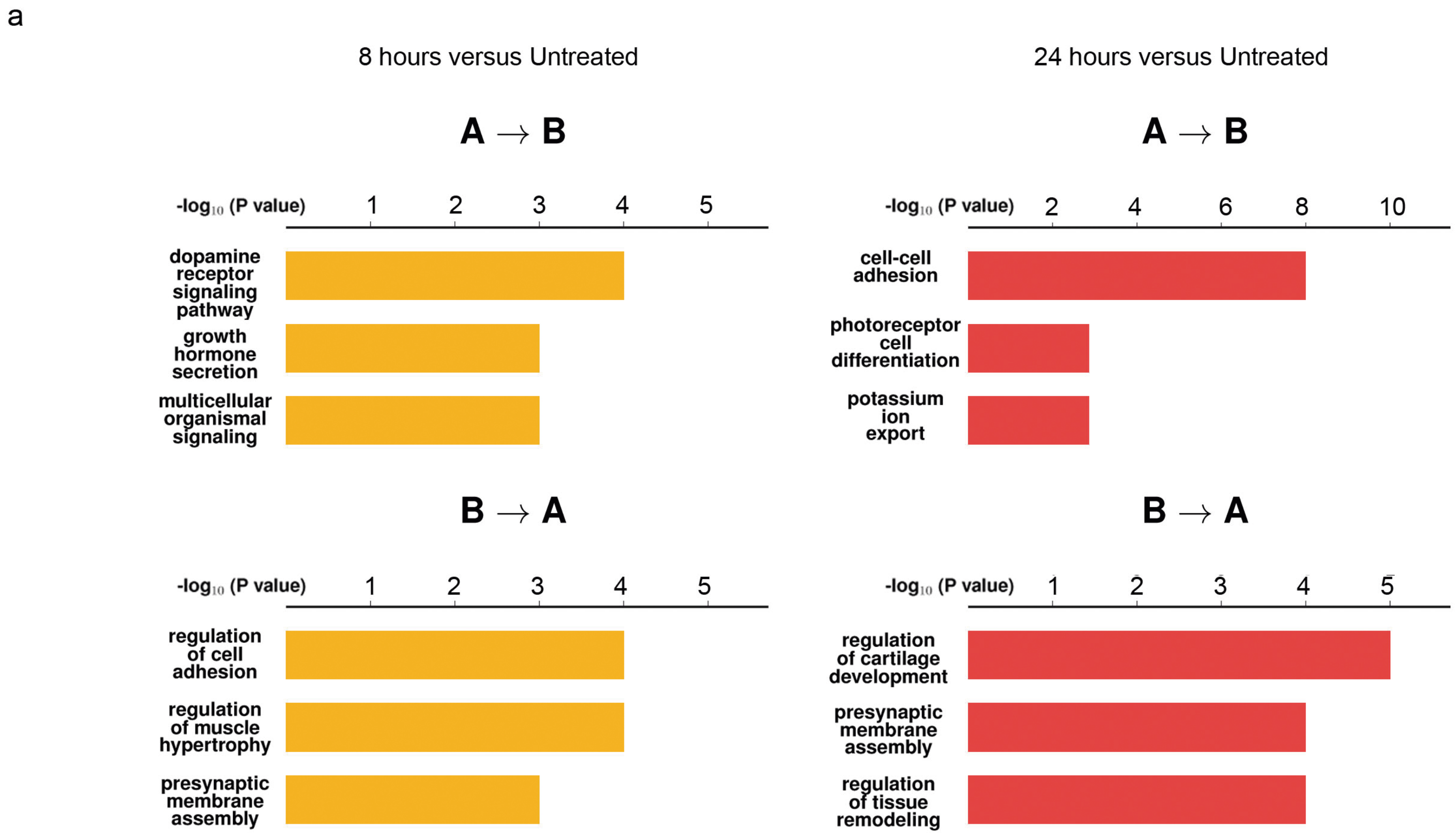

Supplementary Figure 4. Term enrichment analysis of genes that changed compartments. **a** Functional analysis of genes that changed compartment from A to B, or from B to A, at 8 h or 24 h after TGF-β treatment, as compared to untreated NMuMG cells. Only statistically significant categories are depicted.

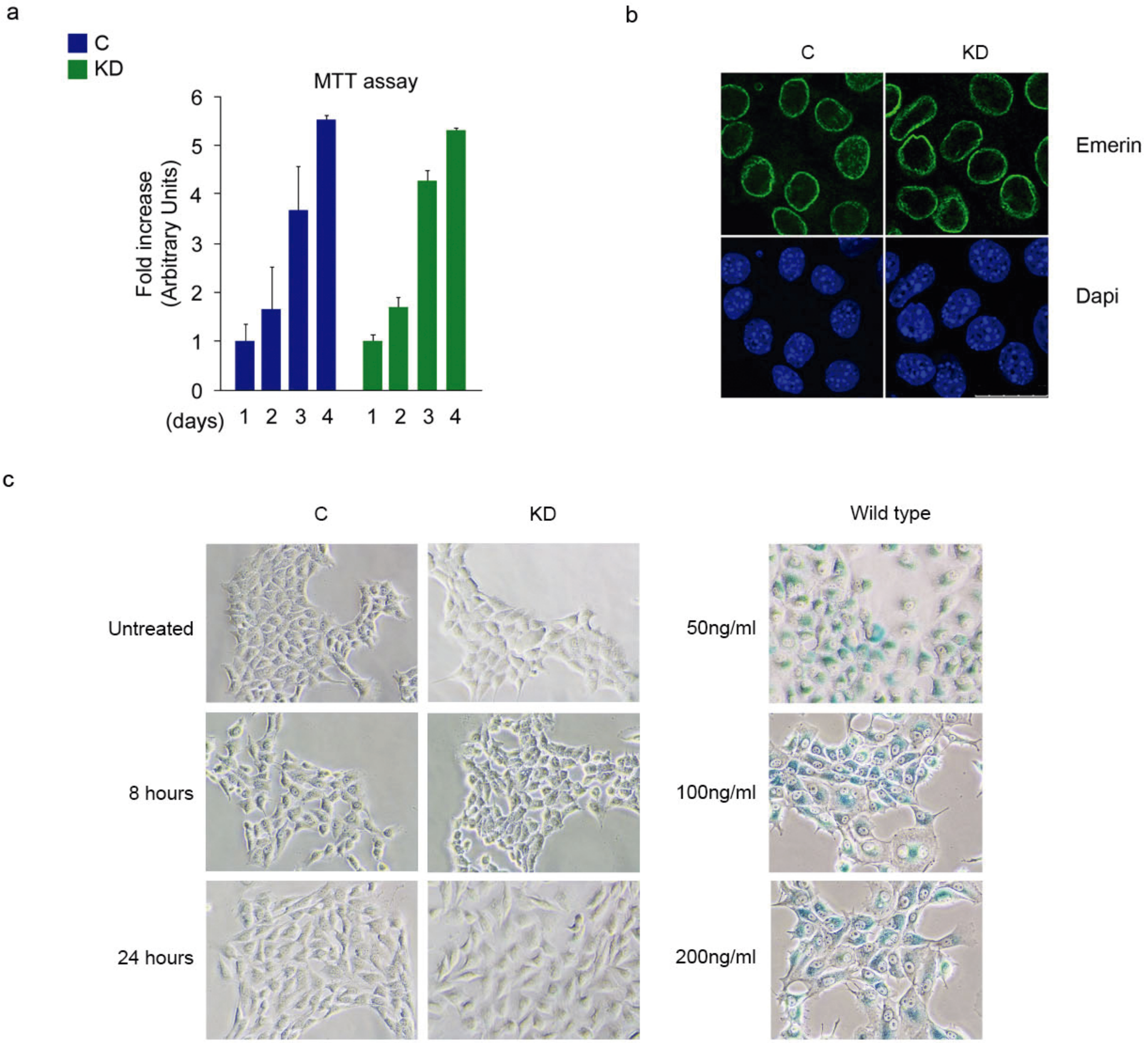

Supplementary Figure 5. Characterization of NMuMG cells knockdown for lamin B1. **a** Quantification of cell viability by the MTT assay in NMuMG cells infected with either control (C) or lamin B1 KD. Measurements were obtained over four consecutive days in three independent replicates. Data are represented as the fold-change relative to day 1, which was set as 1. Data are shown as mean SD. **b** C and KD NMuMG cells were immunolabelled with an antibody against emerin (a nuclear envelope protein) and stained with DAPI. Scale bar, 25 m. c SA-β−gal (senescence-associated galactosidase) staining of C and KD NMuMG cells that were untreated or treated with TGF-β for 8 h or 24 h (left). SA-β−gal staining of wild-type NMuMG cells treated with different concentrations of doxorubicin for 48 h was used as a positiive control of senescence (right). Images are representative of three independent replicates.

a

1041 up-regulated genes in KD cells upon 8 hours TGFβ treatment

Gene Set Name [# Genes (K)]	Description	# Genes in Overlap (k)	k/K	p-value	FDR q-value
HALLMARK_MTORC1_SIGNALING [200]	Genes up-regulated through activation of mTORC1 complex.	20		$2.49\ e^{-9}$	$1.25\ e^{-7}$
HALLMARK_HEME_METABOLISM [200]	Genes involved in metabolism of heme (a cofactor consisting of iron and porphyrin) and erythroblast differentiation.	16		$1.97\ e^{-6}$	$1.68\ e^{-5}$
HALLMARK_MITOTIC_SPINDLE [200]	Genes important for mitotic spindle assembly.	16		$1.97\ e^{-6}$	$1.68\ e^{-5}$
HALLMARK_TNFA_SIGNALING_VIA_NFKB [200]	Genes regulated by NF-kB in response to TNF [GeneID=7124].	16		$1.97\ e^{-6}$	$1.68\ e^{-5}$
HALLMARK_UNFOLDED_PROTEIN_RESPONSE [113]	Genes up-regulated during unfolded protein response, a cellular stress response related to the endoplasmic reticulum.	12		$2.02\ e^{-6}$	$1.68\ e^{-5}$
HALLMARK_ADIPOGENESIS [200]	Genes up-regulated during adipocyte differentiation (adipogenesis).	15		$8.96\ e^{-6}$	$5.6\ e^{-5}$

Supplementary Figure 6. Term enrichment analysis of genes upregulated in KD NMuMG cells after 8 h of TGF-β treatment. Statistically significant functional analysis of 1 041 genes upregulated in KD conditions at 8 h after TGF-β treatment.

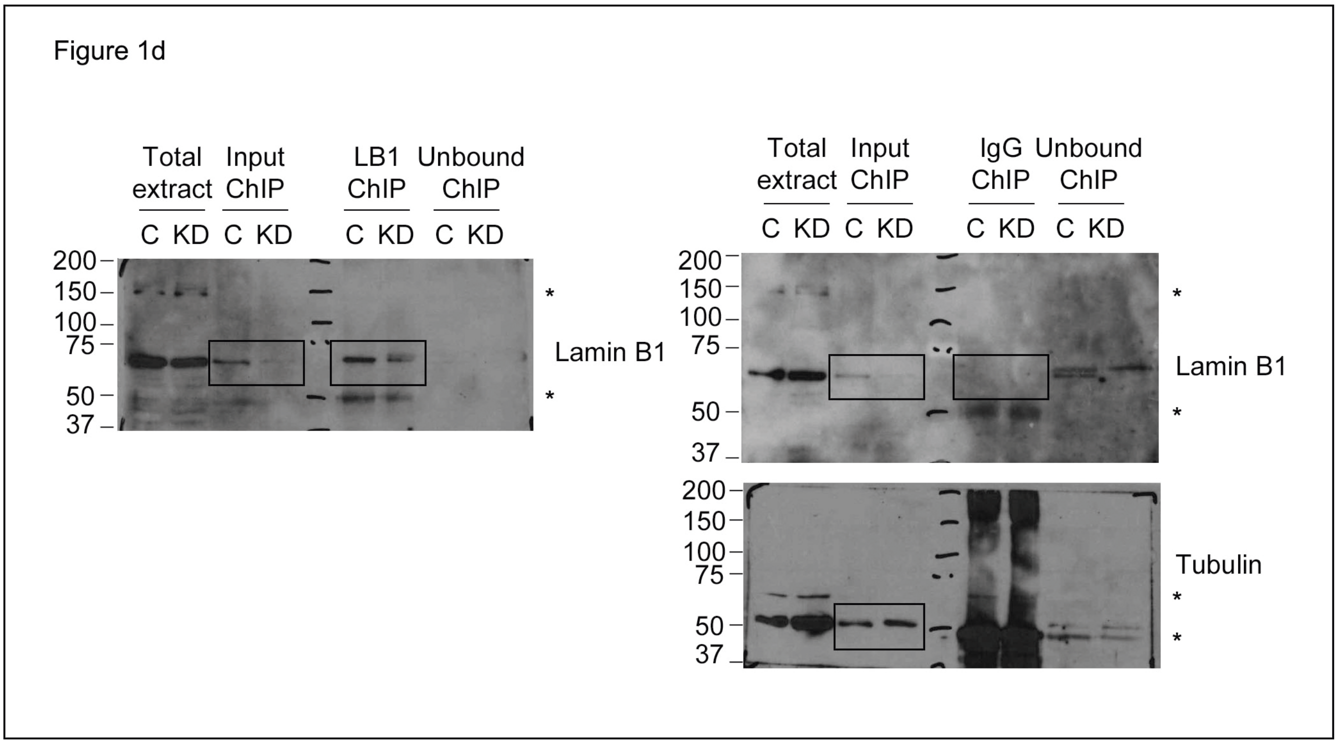

Supplementary Figure 7. Full scans of Western Blots from Figure 1d. Asterisks denote unspecific bands.

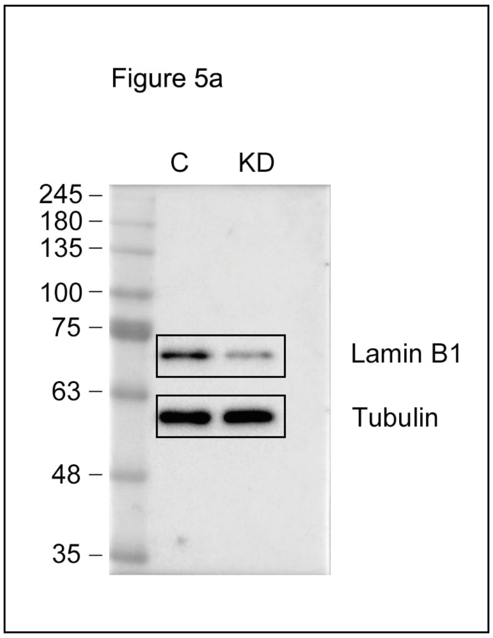

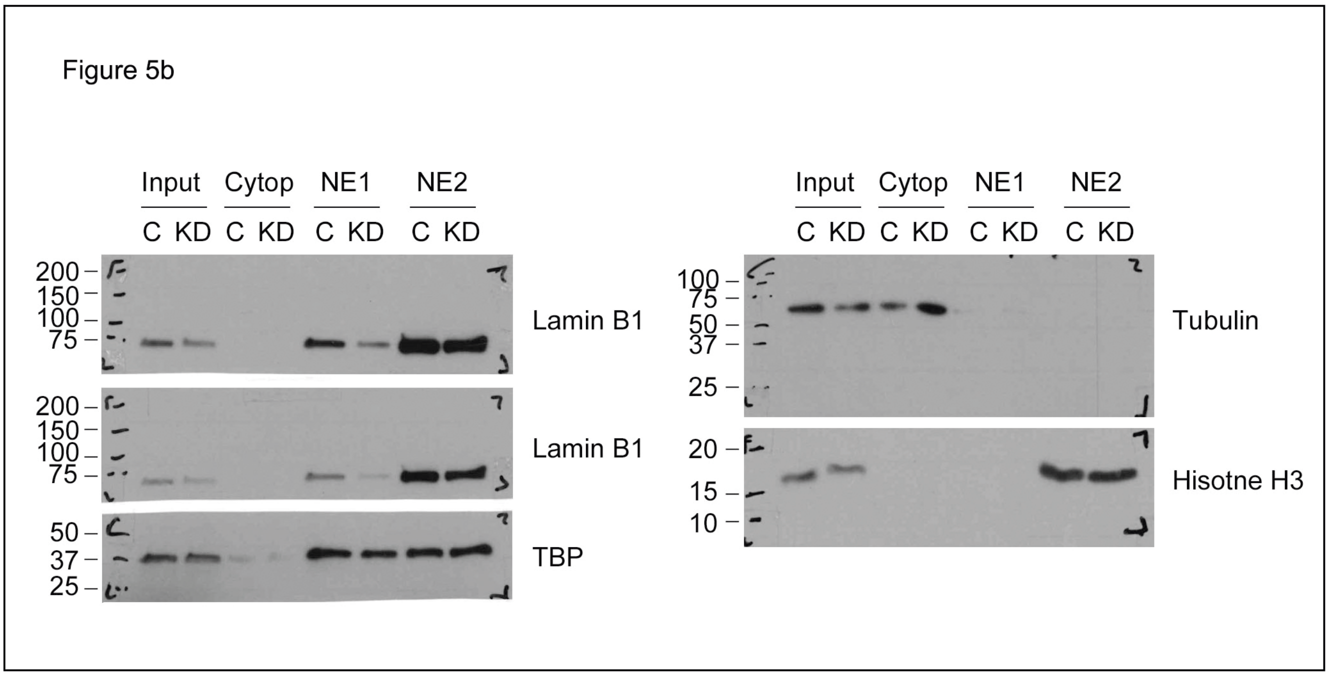

Supplementary Figure 8. Full scans of Western Blots from Figure 5a and 5b.

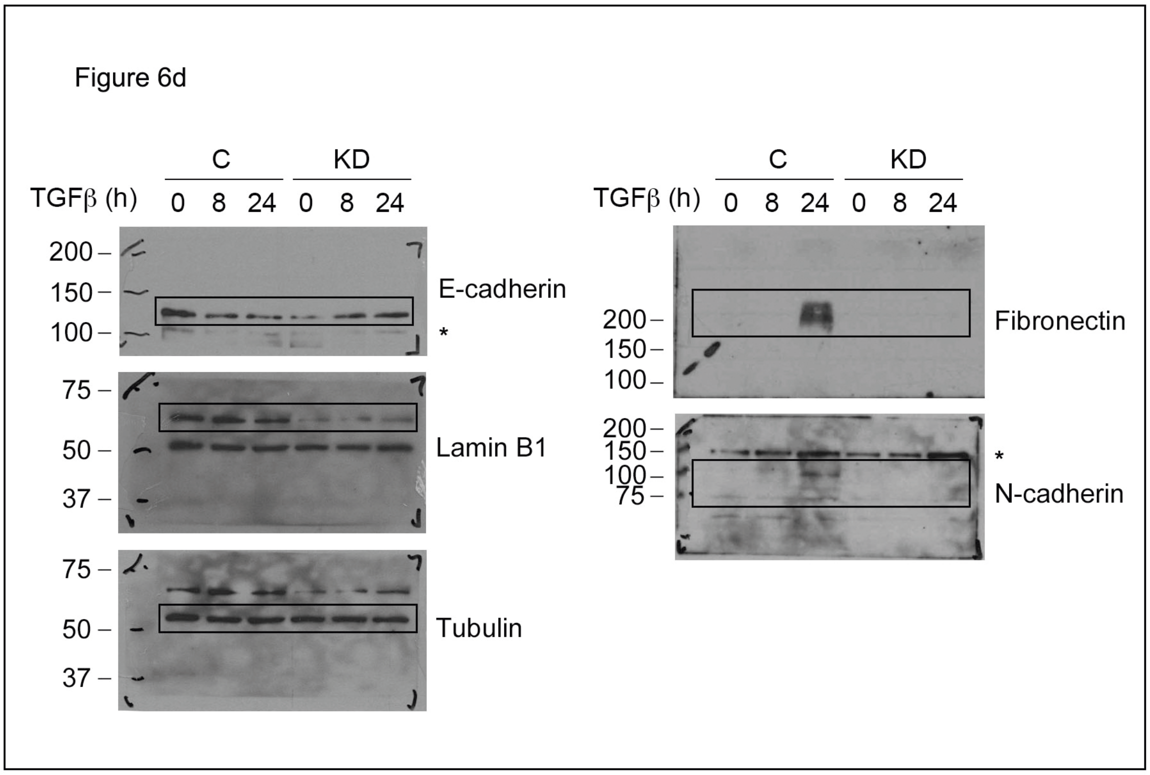

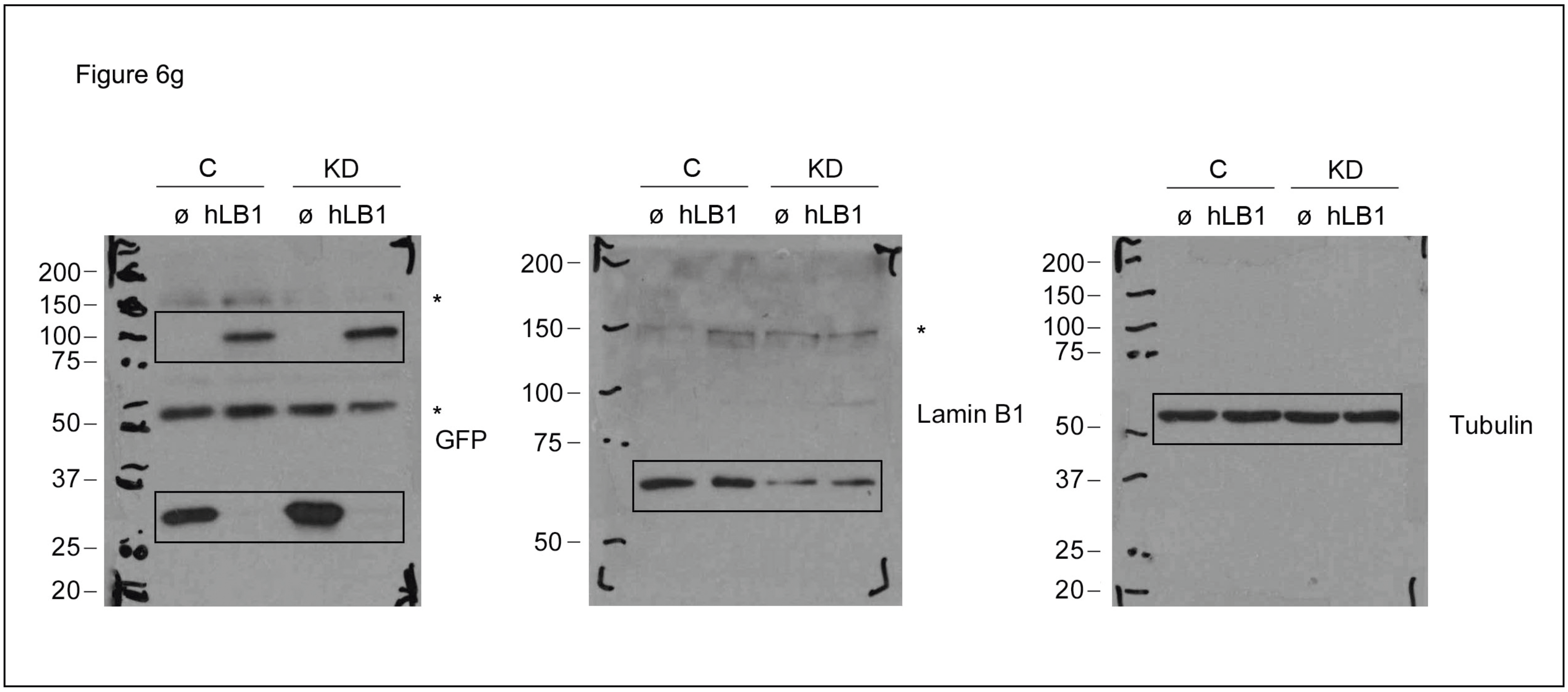

Supplementary Figure 9. Full scans of Western Blots from Figure 6d and 6g. Asterisks denote unspecific bands.

ASCL1
E2F2
Egr1
Ehf
FOXC2
GATA3
Glis2
HOXA13
Hoxd13
KLF5
Klf7
MZF1
Meis1
Mtf1
Myog
NFATC1
NFIC
NHLH1
Nkx3-1
Nr2f2
OLIG3
PROX1
REST
RUNX3
SP1
SP2
Sfpi1
Six6
Smad4
Srf
Tcf12
XBP1
YY1
YY2
ZIC1
ZNF354C
ZNF384
Zbtb7b
Zfp161
Zfp281

Supplementary Table 1. Full list of transcription factors enriched in 5 896 *de novo* lamin B1+ genes

Supplementary Table 2

Sample (and read)	Reads	Mapped reads	%	Mapped Int.	%
**0h read 1**	211679877	185780142	87,76		
**0h read 2**	211679877	179014320	84,57	163087664	77,04
**8h read 1**	196125007	171021097	87,20		
**8h read 2**	196125007	166820416	85,06	152109607	77,56
**24h read 1**	182595645	159768798	87,50		
**24h read 2**	182595645	152521578	83,53	138758327	75,99

Supplementary Table 2

Valid int.	%	Self circle	%	Extra-dangling end	Dangling
88644054	54,35	140238	0,086	37409305	16072737
105589112	69,42	134342	0,088	28550773	23005977
90046184	64,89	95052	0,069	25232513	19243994

Supplementary Table 2

%	Duplicates	%	Error	%	Over rep.	%
32,79	65746047	40,31	1070645	0,66	2614142	1,60
33,89	28381515	18,66	569230	0,37	2613689	1,72
32,05	42420520	30,57	590078	0,43	2144698	1,55

Too short	%	Too large	%	Random breaks	%
14837707	9,10	2634	0,0	702994	0,43
12351009	8,12	3034	0,0	1377994	0,91
12943419	9,33	2105	0,0	1077308	0,78

Supplementary Table 2. Hi-C sequencing data

Time	A	B	eLAD	A+eLAD	% inc	Exp Genes
0	21534	23720	15822	13049	0%	7972
8	21660	23839	19273	13774	6%	8493
24	23463	22872	24220	15144	16%	8170

Time	A	B	eLAD	B+eLAD	% inc	Exp Genes
0	21534	23720	15822	1418	0%	189
8	21660	23839	19273	3714	162%	271
24	23463	22872	24220	7123	402%	391

Supplementary Table 3. *De novo* formation of eLADs in the A or B compartment during EMT

Primer	Direction	Sequence	Application
LAD region	Forward	5’-CAAGCTGCACTGGGACAAAG-3’	ChIP-qPCR
LAD region	Reverse	5’-CAAATGTATGGTGTCCTGAAGGTT-3’	ChIP-qPCR
LAD 1	Forward	5’-TCCATGGGTGACAGGGAC-3’	ChIP-qPCR
LAD 1	Reverse	5’-TCTTTGGGCATCATTTGCTT-3’	ChIP-qPCR
LAD 2	Forward	5’-CCAAAGCTGTTCAGTGAGAGG-3’	ChIP-qPCR
LAD 2	Reverse	5’-CGATAGGGAAGACAGGAGACAC-3’	ChIP-qPCR
LAD 3	Forward	5’-CAAGCTGCACTGGGACAAAG-3’	ChIP-qPCR
LAD 3	Reverse	5’-CAAATGTATGGTGTCCTGAAGGTT-3’	ChIP-qPCR
eLAD 1	Forward	5’-TGCTCTCTCCCTTTGGACC-3’	ChIP-qPCR
eLAD 1	Reverse	5’-CGGGGGTAGGGCATCATAT-3’	ChIP-qPCR
eLAD 2	Forward	5’-GACGTCTTGTGACCGGGTT-3’	ChIP-qPCR
eLAD 2	Reverse	5’-CGGCAGCAGTAGGAGCAGT-3’	ChIP-qPCR
eLAD 3	Forward	5’-GCGCCCGTCGTCCTTCTCGTC-3’	ChIP-qPCR
eLAD 3	Reverse	5’-CTTCCGCGACTGGGGGTCCT-3’	ChIP-qPCR
Fibronectin	Forward	5’-TGAGCATCTTGAGTGGATGG-3’	ChIP-qPCR
Fibronectin	Reverse	5’-GTGTGAGCCGGACAACTTCT-3’	ChIP-qPCR
Zeb2	Forward	5’-GGGCCTCTTCTTACCGTTTT-3’	ChIP-qPCR
Zeb2	Reverse	5’-CGCTGTGTTTGGTTGCTAGA-3’	ChIP-qPCR
Twist2	Forward	5’-GCCTCGAAATCAGAGCCTTT-3’	ChIP-qPCR
Twist2	Reverse	5’-TCCAGCTCTTCCTCACTGGT-3’	ChIP-qPCR
prOct4	Forward	5’-ACCAACCTGGACAACACAAGATG-3’	ChIP-qPCR
prOct4	Reverse	5’-GCTTACCCACCCGTCTAGAGTCC-3’	ChIP-qPCR
Lamin B1	Forward	5’-CTGCTGCTCAATTATGCCAAGAAG-3’	qRT-PCR
Lamin B1	Reverse	5’-GGCAGATAAGGATGCTTCTAGCT-3’	qRT-PCR
Pumilio	Forward	5’-CGGTCGTCCTGAGGATAAAA-3’	qRT-PCR
Pumilio	Reverse	5’-CGTACGTGAGGCGTGAGTAA-3’	qRT-PCR

Supplementary Table 4. List of primers used in the manuscript.

Supplementary methods

Cell lines, transfections, and infections

HEK293T cells (ATCC No.: CRL-3216) and NMuMG cells (ATCC No.: CRL-1636) were maintained in Dulbecco's modified Eagle's medium (Invitrogen) with 10% fetal bovine serum (Invitrogen) at 37ºC in 5% CO_2. NMuMG cells were also supplemented with 10 µg/mL insulin (Sigma) at 37ºC in 5% CO_2. TGF-β was added to a final concentration of 1–5 ng/mL to induce EMT in NMuMG cells. All cell lines were regularly tested for the absence of mycoplasma using standard PCR with the following primers: F: 5′-GGCGAATGGGTGAGTAACACG-3′ and R: 5'-CGGATAACGCTTGCGACCTATG-3′.

For lentiviral infections, HEK293T cells were grown to 70% confluency and then transfected (day 0) by adding, drop-wise, a mixture of NaCl, DNA (7.5 µg of mouse shLB1 [TRCN0000091906] or human shLB1 [TRCN0000297205]/ shCT, 1.5 µg pCMV-VSVG, 4.5 µg pMDLg/pRRE and 1.5 µg pRSV rev) and polyethylenimine polymer (Polysciences Inc.) that had been pre-incubated for 15 min at room temperature. At 24 and 48 h after transfection (days 1 and 2), transfection medium was replaced with fresh medium, and the removed transfection medium was filtered with 0.45 µm filter unit (Merck Millipore) and stored at 4ºC (and mixed after day 2). The mixture was concentrated using Lenti-X Concentrator product (Clonetech) and centrifugated at 1500 g for 45 min at 4ºC. The pellet was resuspended in 1 mL fresh medium, aliquoted in 100 µL and stored at –20ºC. NMuMG cells were infected with a single aliquot of the concentrated virus. Infected cells were selected with puromycin for 48 h (1µg/mL). For rescue experiment, NMuMG-infected cells with were seeded 48 h after selection for transfection with Lipofectamine 2000 reagent (Invitrogen) with either a GFP empty vector or human mPA-GFP-LaminB1-10. The transfection medium was replaced with fresh medium after 24 h (day 1). At 48 h after transfection, cells were selected with G418 sulfate (Merk Millipore) for 10 days at a final concentration of 0.5 µg/µL, together with puromycin (1 µg/mL) to maintain the shRNA of LB1.

Antibodies

The following antibodies were used: anti-lamin B1 (ab16048, Abcam; 1/1000 western blot and immunofluorescence, 10μg/500μg of chromatin), anti-GFP (ab6556, Abcam; 1/2000), anti-tubulin (T9026, Sigma-Aldrich; 1/50000), anti-emerin (emerin-CE-S, Leyca Biosystems; 1/50), anti-fibronectin (A0245, Dako; 1/5000), anti-E-cadherin (610182, BD Transduction Laboratories; 1/2000), anti-N-cadherin (610920, BD Transduction Laboratories; 1/1000), anti-TBP (ab63766, Abcam; 1/2000) and anti-H3 (ab1791, Abcam; 1/10000).

Immunofluoroescence, image acquisition and analysis

Cells were fixed with 4% PFA for 15 min at room temperature and blocked for 1 h with 1% PBS-BSA. Cells were then incubated either at room temperature for 2 h or at 4ºC overnight with the primary antibody, washed 3× with PBS and incubated 1 h at room temperature with the secondary antibody. For immunoflourescence, cells were washed 3× with PBS, incubated 5 min with PBS-DAPI (0.25 μg/mL) to stain cell nuclei and then mounted with fluoromount. Fluorescent images were acquired with a Leica TCS SPE confocal microscope using a Leica DFC300 FX camera and the Leica IM50 software.

Cell extracts

Cell extracts from NMuMG cells were obtained in SDS lysis buffer (2% SDS, 50 mM Tris-HCl and 10% glycerol). Proteins were separated by SDS–polyacrylamide gel electrophoresis, and the proteins were detected with the corresponding antibody.

RNA analysis by quantitative RT-PCR (qRT-PCR)

RNA was extracted with TRIzol reagent (Invitrogen) and retrotranscribed with the Transcriptor First Strand cDNA Synthesis kit (Roche). Real-time quantitative experiments were done in a Light Cycler PCR machine (Roche). Primers used are listed in Supplementary Table 4 (Supplementary Table 4).

Analysis of ChIP-seq data

ChIP-seq samples were mapped against the mm9 mouse genome assembly using BowTie with the option –m 1 to discard those reads that could not be uniquely mapped to just one region[1]. MACS was run with the default parameters but with the shift-size adjusted to 100 bp to perform the peak calling against the corresponding control sample[2]. The genome distribution of each set of peaks was calculated by counting the number of peaks fitted on each class of region according to RefSeq annotations. Distal region was the region within 2.5 Kbp and 0.5 Kbp upstream of the transcription start site (TSS). Proximal region was the region within 0.5 Kbp of the TSS. UTR, untranslated region; CDS, protein coding sequence; intronic regions, introns; and the rest of the genome, intergenic. Peaks that overlapped with more than one genomic feature were proportionally counted the same number of times. Pie charts were generated by calculating the genome distribution of all features in the full genome, and the R caroline package was used to combine the piechart of each set of peaks with the full genome distribution[3]. Each set of target genes was retrieved by matching the ChIP-seq peaks in the region 2.5 Kbp upstream of the TSS until the end of the transcripts as annotated in RefSeq. Reports of functional enrichments of gene ontology and ENCODE ChIP-seq histone marks categories were generated using the EnrichR tool[4] and the Molecular Signatures Database (MSigDB)[5]. Plots showing the average distribution of ChIP-seq reads 2 Kbp around the TSS of each target gene were generated by counting the number of reads for each region according to RefSeq and then averaging the values for the total number of mapped reads of each sample and the total number of genes in the particular gene set. The heatmaps displaying the density of ChIP-seq reads 5 Kb around the TSS of each target gene set were generated by counting the number of reads in this region for each individual gene and normalizing this value with the total number of mapped reads of the sample. Genes on each ChIP heatmap were ranked by the logarithm of the average number of reads in the same genomic region. To generate the lists of eLADs, the signal of the corresponding IgG was subtracted from each LB1 ChIP-seq sample to score each bin of 100 bps along the genome in terms of normalized reads. Next, a threshold of 1 unit was set to filter out those bins without peaks. Finally, two or more individual peaks of LB1

were clustered at a distance less than 0.25 Mb. The UCSC genome browser was used to generate the screenshots of each group of experiments along the manuscript[6].

Analysis of RNA-seq data

The RNA-seq samples were mapped against the mm9 mouse genome assembly using TopHat[7] with the option –g 1 to discard those reads that could not be uniquely mapped in just one region. Cufflinks and Cuffdiff were run to quantify the expression in FPKMs of each annotated transcript in RefSeq and to identify the list of differentially expressed genes for each case[8].

Analysis of ATAC-seq data

The ATAC-seq samples were mapped against the mm9 mouse genome assembly using BowTie with the option –m 1 to discard those reads that could not be uniquely mapped to just one region, and with the option –X 2000 to define the maximum insert size for paired-end alignment[1]. MACS was run with the default parameters but with the shiftsize adjusted to 100 bp to perform peak calling[2]. Each set of target genes was retrieved by matching the ChIP-seq peaks in the region 2.5 Kb upstream of the TSS until the end of the transcripts as annotated in RefSeq. Reports of functional enrichments of gene ontology and ENCODE ChIP-seq histone marks categories were generated with the EnrichR tool[4].

Analysis of Hi-C data

Hi-C data was processed using TADbit[9] for read quality control, read mapping, interaction detection, interaction filtering and matrix normalization. First, reads were checked using an implemented FastQC protocol (http://www.bioinformatics.babraham.ac.uk/projects/fastqc) in TADbit. This allowed problematic samples to be discarded and systematic artefacts to be detected. Next, a fragment-based strategy in TADbit was used to map the remaining reads to the reference mouse genome (reference mm9). The mapping strategy resulted in about 85% of reads mapped uniquely to the genome (Supplementary Table 1). Non-informative contacts between

two reads, including self-circles, dangling-ends, errors, random breaks, or duplicates, were filtered as previously described[9]. The final interaction matrices resulted in 89–106 M of valid interactions per time point (Supplementary Table 1). These valid interactions were then used to generate genome-wide interaction maps at 100, 40 and 5 Kb to segment the genome into the so-called A/B compartments or topologically associating domains (TADs) or to perform a meta-analysis, respectively[10,11]. A/B compartments were calculated using normalized and decay-corrected matrices[12] by calculating the first component of a PCA of chromosome-wide matrices of the Pearson product-moment internal correlation as implemented in HOMMER[13]. TADs were identified using 100 Kb resolution normalized and decay-corrected matrices as input to a series of scripts from the Dekker lab with default parameters[14]. TAD border localization and strength was calculated and used to identify conserved borders and their strengths. A border was considered conserved if it was localized in the same bin in the three experiments. Finally, to assess whether particular parts of the Hi-C interaction matrices had common structural features, a meta-analysis of the region was performed by merging individual local submatrices at 5 Kb resolution in similar fashion as previously published[15]. 4C-like profiles centred at the TSS of several selected genes were extracted from the meta-matrices and compared between untreated and treated samples. The resulting differential 4C-like profiles were calculated by subtracting the treated from the untreated interaction counts, which were represented as line plots or heatmaps.

Supplementary references

1 Langmead, B., Trapnell, C., Pop, M. & Salzberg, S. L. Ultrafast and memory-efficient alignment of short DNA sequences to the human genome. *Genome Biol* **10**, R25, doi:10.1186/gb-2009-10-3-r25 (2009).

2 Zhang, Y. *et al.* Model-based analysis of ChIP-Seq (MACS). *Genome Biol* **9**, R137, doi:10.1186/gb-2008-9-9-r137 (2008).

3 Feitelson, D. G. Comparing Partitions with Spie Charts. *Technical Report* 2003-2087 (2003).

4 Kuleshov, M. V. *et al.* Enrichr: a comprehensive gene set enrichment analysis web server 2016 update. *Nucleic Acids Res* **44**, W90-97, doi:10.1093/nar/gkw377 (2016).

5 Liberzon, A. *et al.* Molecular signatures database (MSigDB) 3.0. *Bioinformatics (Oxford, England)* **27**, 1739-1740, doi:10.1093/bioinformatics/btr260 (2011).

6 Kent, W. J. *et al.* The human genome browser at UCSC. *Genome Res* **12**, 996-1006, doi:10.1101/gr.229102. Article published online before print in May 2002 (2002).

7 Trapnell, C., Pachter, L. & Salzberg, S. L. TopHat: discovering splice junctions with RNA-Seq. *Bioinformatics* **25**, 1105-1111, doi:10.1093/bioinformatics/btp120 (2009).

8 Roberts, A., Trapnell, C., Donaghey, J., Rinn, J. L. & Pachter, L. Improving RNA-Seq expression estimates by correcting for fragment bias. *Genome Biol* **12**, R22, doi:10.1186/gb-2011-12-3-r22 (2011).

9 François Serra, D. B., Guillaume Filion, Marc A. Marti-Renom. Structural features of the fly chromatin colors revealed by automatic three-dimensional modeling. *bioRxiv*, doi:doi.org/10.1101/036764 (2016).

10 Lieberman-Aiden, E. *et al.* Comprehensive mapping of long-range interactions reveals folding principles of the human genome. *Science* **326**, 289-293, doi:10.1126/science.1181369 (2009).

11 Nora, E. P. *et al.* Spatial partitioning of the regulatory landscape of the X-inactivation centre. *Nature* **485**, 381-385, doi:10.1038/nature11049 (2012).

12 Imakaev, M. *et al.* Iterative correction of Hi-C data reveals hallmarks of chromosome organization. *Nat Methods* **9**, 999-1003, doi:10.1038/nmeth.2148 (2012).

13 Heinz, S. *et al.* Simple combinations of lineage-determining transcription factors prime cis-regulatory elements required for macrophage and B cell identities. *Molecular cell* **38**, 576-589, doi:10.1016/j.molcel.2010.05.004 (2010).

14 Lajoie, B. R., Dekker, J. & Kaplan, N. The Hitchhiker's guide to Hi-C analysis: practical guidelines. *Methods* **72**, 65-75, doi:10.1016/j.ymeth.2014.10.031 (2015).

15 de Wit, E. *et al.* The pluripotent genome in three dimensions is shaped around pluripotency factors. *Nature* **501**, 227-231, doi:10.1038/nature12420 (2013).
